# Comparison of Effects of ACEIs and ARBs on Albuminuria and Hyperkalemia in Indonesian Hypertensive Type 2 Diabetes Mellitus Patients

**DOI:** 10.1155/2020/5342161

**Published:** 2020-07-29

**Authors:** Putri S. Agustina, Em Yunir, Pukovisa Prawiroharjo, Johanda Damanik, Rani Sauriasari

**Affiliations:** ^1^Faculty of Pharmacy, Universitas Indonesia, Depok, Indonesia; ^2^Department of Internal Medicine, Faculty of Medicine, Universitas Indonesia, Jakarta, Indonesia; ^3^Department of Neurology, Faculty of Medicine, Universitas Indonesia, Jakarta, Indonesia

## Abstract

**Purpose:**

Due to economic consideration, Indonesia's formulary restrictions are at odds with the treatment guidelines of the American Diabetes Association (ADA) and the Eighth Joint National Committee (JNC 8). ADA and JNC 8 equally recommend the prescription of an angiotensin-converting enzyme inhibitor (ACEI) or angiotensin II receptor blocker (ARB) for hypertensive patients with type 2 diabetes mellitus (T2DM) with overt proteinuria (urine albumin to creatinine ratio (UACR) ≥ 300 mg/g creatinine). However, since 1 April 2018, Indonesian formulary restricted telmisartan and valsartan only for T2DM patients with declined renal function as shown by eGFR value. There is no compelling evidence in favor of ACEI over ARB or vice versa except for data supporting the early use of both drugs in patients with overt proteinuria. However, ARB is a choice if ACEI's side effects, that is, coughing, occurs. Therefore, it necessitates a detailed evaluation of the effects of ACEIs and ARBs on albuminuria and their side effect, hyperkalemia, specific to Indonesian T2DM patients.

**Methods:**

This cross-sectional study involved 134 T2DM patients whose treatment was restricted to either ACEIs (*n* = 57) or ARBs (*n* = 77) for at least two months before the study during May–October 2018. Patients with known end-stage renal disease and those receiving dialysis were excluded. UACR and blood potassium levels were compared between the two study groups. Also, the risk factors of albuminuria and hyperkalemia were estimated using multivariate analysis.

**Results:**

T2DM patients in the ACEI and ARB groups had similar characteristics except for a higher body mass index (*p*=0.008), lower glomerular filtration rate (*p*=0.04), and a longer duration of prior treatment (*p* < 0.001) in the ARB group. This study showed no differences between the ACEI and ARB groups in the proportion of cases with albuminuria (*p*=0.97) and hyperkalemia (*p*=0.86), even after adjustment for confounders. In addition, uncontrolled diastolic blood pressure was a significant factor associated with albuminuria (OR: 4.897, 95% CI: 1.026–23.366; *p*=0.046), whereas a female was 70.1% less likely to develop hyperkalemia than a male (OR: 0.299, 95% CI: 0.102–0.877; *p*=0.028).

**Conclusion:**

This cross-sectional study demonstrated that ACEIs and ARBs have a similar effect on albuminuria and hyperkalemia in Indonesian hypertensive T2DM patients, even after correction for potentially confounding variables.

## 1. Introduction

Chronic hyperglycemia in diabetes mellitus is associated with various complications, including retinopathy, neuropathy, and nephropathy. Glomerulosclerosis, that is, thickening of the glomerular basement membrane, glomerular hypertrophy, increased proliferation of mesangial cells, loss of podocytes, and tubulointerstitial fibrosis are responsible for impairments in kidney functioning that may culminate in diabetic nephropathy [1]. These cellular changes also prompt the development of albuminuria, declining glomerulus filtration rate, increased arterial blood pressure, and increased renal flow resistance [1]. Around 40% of diabetic patients are reported to develop diabetic nephropathy [2]. According to a survey by the Indonesian Society of Nephrology (PERNEFRI) in 2009, 12.5% of the Indonesian population experienced renal dysfunction due to a variety of causes such as hypertension, diabetes, lupus, and polycystic kidney disease [3]^.^

Antihypertensive therapeutics inhibiting the renin–angiotensin–aldosterone (RAS) system, such as angiotensin-converting enzyme inhibitors (ACEIs) and angiotensin receptor blockers (ARBs), are generally recommended for diabetic and nondiabetic patients when used together with calcium channel blockers and thiazide diuretics. Moreover, based on American Diabetes Association ADA guidelines (2018), ACEIs and ARBs are the only two drug classes recommended for hypertensive type 2 diabetes mellitus (T2DM) patients with albuminuria, to slow down the development of chronic kidney disease [4]. Decreased risk of renal failure was reported in patients with diabetic nephropathy who received ACEIs. Furthermore, the administration of the maximum tolerable dose of ACEIs reduced mortality in these patients [5]. ARBs were also reported to reduce proteinuria and blood pressure in diabetic patients with nephropathy and hypertension [6]. The renoprotective effects of ACEIs or ARBs in diabetic patients are reflected in decreased progression of macroalbuminuria and increased occurrence of normoalbuminuria and microalbuminuria [7]. The different mechanisms of action of the two classes of therapeutics might result in different renoprotective effects [8]. Telmisartan, an ARB, acts as a partial PPAR-*γ* agonist, which limits inflammation induced by high glucose concentrations in proximal tubular cells [9]. Meanwhile, Hsu et al. reported that ACEIs provide better renoprotective effects, with a better safety profile than that of ARBs [10]. A single administration of ACEIs or ARBs as the first-line treatment reduces diabetic nephropathy progression, although combined treatment with ACEIs and ARBs increases the risk of hyperkalemia without providing additional clinical benefits [11].

Research on ACEIs and ARBs still lacks in Indonesia. Suhadi et al. reported that hypertensive diabetic patients demonstrated no significant differences in renoprotective effects between single or combined antihypertensive therapy with or without RAS inhibitors [12]. A study by Helmidanora et al. showed that diabetic hypertensive patients receiving either ACEIs or ARBs as antihypertensive monotherapy did not show different renoprotective effects, as evaluated by qualitative measurements of the severity proteinuria levels [13].

American Diabetes Association (ADA) and Eighth Joint National Committee (JNC 8) equally recommend the prescription of ACEI or ARB for hypertensive patients with T2DM with overt proteinuria (UACR ≥ 300 mg/g creatinine) [4]. However, due to economic consideration, formulary restrictions in Indonesia are at odds with the treatment guidelines of ADA (2018) and JNC 8. Since April 2018, the study location effectively adopted national formulary restrictions including the following:

1. All dosage forms of valsartan and telmisartan should be accompanied by laboratory results showing that the patient's estimated glomerular filtration rate (eGFR) was <60 ml/min/1.73 m^2^.
2. Candesartan and irbesartan administration is only permitted if accompanied by a written statement by the prescribing doctor, stating that the patient is resistant to ACEI [14].

There is no compelling evidence in favor of ACEI over ARB or vice versa except for data supporting the early use of both drugs in patients with overt proteinuria. However, ARB is a choice if ACEI's side effects, that is, coughing, occurs. Since there has been a paucity of research on the efficacy of ACEIs and ARBs for renoprotection in Indonesian diabetic hypertensive patients, insufficient evidence was available to judge whether ACEIs or ARBs might be the more effective renoprotective agents. Therefore, we conducted this research to get more detailed comparisons of the efficacies of ACEIs and ARBs on albuminuria and also their side effect, hyperkalemia, specific to Indonesian T2DM patients.

## 2. Materials and Methods

The present, preliminary, cross-sectional study was conducted as part of the larger cohort study “Assessment of Renal Function and Cognitive Function in Type 2 Diabetic Patients: Parameters for Decreasing Organ Functions.” This study was conducted at RSUPN Dr. Cipto Mangunkusumo Hospital, Jakarta, Indonesia, the National Referral Center for Government Hospitals in Indonesia, and serves as a teaching hospital. The period of study was more than six months in 2018.

### 2.1. Patients

All of the participants were outpatients of diabetes polyclinic at the RSUPN Dr. Cipto Mangunkusumo, Hospital, Jakarta. Diabetes was defined based on the clinician's judgment. The minimum numbers of participants were calculated by the formula

(1) $$\begin{array}{c} n=\frac{{\left(Z_{\alpha /2}+Z_{\beta }\right)}^{2}\times 2\sigma^{2}}{{\left({µ}_{1}-{µ}_{2}\right)}^{2}},\\ n=\frac{{\left(1.96+0.84\right)}^{2\;}\times 2{\left(52\right)}^{2}}{{\left(28\right)}^{2}},\\ n=54. \end{array}$$

Thus, the minimally required number of participants in each treatment group was 54 patients. A total of 134 type 2 diabetic patients were enrolled in the study, 57 of whom received ACEIs and 77 of whom received ARB for at least two months prior the study. Patients were excluded from participation when diagnosed with end-stage renal disease (ESRD), when receiving dialysis treatment or corticosteroid therapy or when taking contraceptives. Finally, anemic and pregnant patients were excluded from the present study.

### 2.2. Study Approval and Informed Consent

This study was reviewed and approved by the Ethics Committee of the Universitas Indonesia–Dr. Cipto Mangunkusumo Hospital (No. 222/UN2.F1/ETIK/2018), and all procedures involving human subjects were in accordance with the Helsinki Declaration of 1975 (revised in 2008). All subjects were aged over 18 years and agreed to participate in the study procedures by signing an informed consent form.

### 2.3. Clinical Data Collection

The collected information included data on demographics, socioeconomic status (i.e., level of education and employment status), medical history (including a family history of diabetes, obesity, hypertension, and dyslipidemia), smoking status, comorbidities, and concurrent medications and medication adherence, through the Morisky, Green, and Levine (MGL) adherence questionnaire [15]. Patients were asked whether they were aware of their hypertensive state as well as their receiving of ACEIs or ARBs before enrolment in this study. Predetermined clinical and laboratory data included HbA1c, fasting serum glucose, and serum lipid profile (total cholesterol, low-density lipoprotein, high-density lipoprotein, and triglycerides), which were retrieved from patients' electronic medical records at the RSUPN Dr. Cipto Mangunkusumo Hospital. Laboratory measurements of serum creatinine, serum potassium, urine creatinine, urine albumin, and albumin to creatinine ratio (ACR) were obtained within three months before or one month after screening in the pathology laboratory of the RSUPN Dr. Ciptomangukusumo Hospital. The eGFR was calculated using the CKD-EPI study equation [4]. Albuminuria was defined as UACR >30 mg/g creatinine, whereas hyperkalemia was defined if serum potassium level was ≥5.5 mmol/L.

Blood samples were collected on the spot, and urine samples were collected early in the morning. Serum creatinine (mg/dl) was determined by an ARCHITECT c8000 Clinical Chemistry Analyzer (Abbot, USA). ACR was measured by a NycoCard^™^ U-Albumin (Abbot, USA).

## 3. Statistical Analysis

Demographic and clinical information recorded at baseline were analyzed using IBM^®^ SPSS^®^ statistical software, version 23. Kolmogorov–Smirnov tests were performed to examine group distributions of data. Continuous data were expressed in mean ± SD if normally distributed or median (min-max) if not normally distributed. Categorical variables were expressed as frequency ranges with percentages and were compared using chi-square tests. Logistic regression multivariate analysis with odds ratios was applied to examine the associations between patient characteristics and treatment outcomes, that is, albuminuria and hyperkalemia. Covariates were analyzed using chi-square tests, and variables with *p* < 0.25 and the variables considered relevant in the literature were analyzed by multivariate analysis. The selected variables were analyzed by the binary logistic regression backward likelihood ratio method, and the tests with *p* values < 0.05 were regarded as statistically significant.

## 4. Results and Discussion

### 4.1. Results

**Figure 1:**
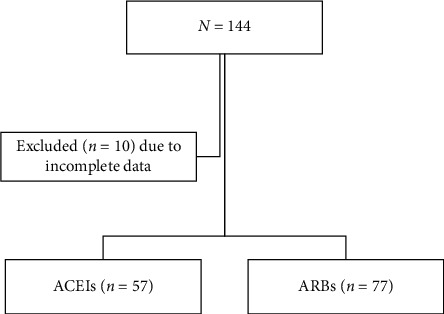
Recruitment process. ACEIs, angiotensin converting enzyme inhibitors; ARBs, angiotensin II receptor blockers.

Of the 134 patients that met the inclusion criteria and enrolled in this study, 77 (57.5%) patients were on ARBs medication. The most age group in the ARB group was 51–60 years old (Figure 1). The number of participants in the ACEI-treated and the ARB-treated groups showed apparent differences due to the application of the inclusion and exclusion criteria. However, both groups met the minimum, statistically required number of individuals. Most of the T2DM patients using ACEIs or ARBs were unemployed and were high school graduates. A majority of patients had been diagnosed with diabetes for more than five years before. Most of the patients were nonsmokers, whereas only a few of them were ex-smokers (Table 1).

Well-known, crucial factors that lead to complications of T2DM include having a long history of diabetes, dyslipidemia, and elevated HbA1c levels. These factors may constitute a higher risk for the development of chronic complications. Most of the patients were found to maintain poor glycaemic control. Additionally, bivariate analysis of HbA1c levels in the ACEI and ARB groups did not show significant between-group differences. In addition, logistic regression, multivariate analysis to examine associations between HbA1c and treatment outcomes (albuminuria and hyperkalemia) did not show significant effects on the efficacy of the two compared drug therapies. Furthermore, the majority of the patients had LDL cholesterol levels above 100 mg/dL (Table 1).

Patients received various antidiabetic agents, especially combinations of oral diabetic drugs and insulin. Besides ACEIs or ARB, most of the participating patients received other antihypertensive drugs. The majority of patients were being treated with ACEIs or ARBs and one non-RAS inhibitor as antihypertensive drugs. In addition, most patients had been receiving ARBs for more than 6 months. Adherence to treatment, as evaluated by the MGL medication adherence questionnaire, showed moderate medication adherence among the patients (Table 1). Overall, most characteristics of diabetic patients on ACEIs or ARBs were equally distributed, except for BMI and duration of ACEI or ARB treatment.

Patients with uncontrolled diastolic blood pressure had albuminuria more often than patients with controlled diastolic blood pressure (Table 2). Among female patients, there were fewer cases of albuminuria than among males (*p*=0.068). ACEIs or ARBs treatment did not differ in their effects on the occurrence of hyperkalemia (*p* > 0.05) (Table 3). Among patients with moderate therapeutic adherence, there were relatively fewer hyperkalemia cases than among highly adherent patients, although this effect was statistically insignificant (*p*=0.136) (Table 3).

### 4.2. Discussion

The pathophysiology of renal dysfunction in diabetic patients develops via metabolic and hemodynamic pathways [1]. The current CKD guidelines recommend the use of ACEIs/ARBs as antiproteinuric drugs, despite the antihypertensive effects of both drug classes (4.16). Regardless of reducing blood pressure, angiotensin 2 is more harmful in renal dysfunction because of the constricting ability of efferent arterioles [16]. Activation of the angiotensin 2 receptor is hypothesized to antagonize detrimental effects of AT1 receptor activation, such as increased oxidative stress, growth factor, cytokines, chemokines, and preinflammation and fibrogenic mediators [16].

The patient characteristics of the ACEI- and ARB-treated patients groups in this study were equal except for BMI and the duration of RAS inhibitor treatment. ARB-treated patients have a larger overall BMI than ACE-treated patients. The majority of T2DM patients on ARBs received insulin treatment combined with an oral antidiabetic agent. Patients who receive insulin treatment generally report increasing body weight [17]. Body weight increments are related to insulin therapy caused by low blood glucose concentration that reaches renal threshold without low-calorie intake compensation, unconscious high-calorie intake to prevent hypoglycaemia, and pharmacokinetic and metabolic profiles due to subcutaneous administration [18]. Differences in duration of RAS inhibitor therapy in both groups show that ARB group experiences less antihypertensive therapy change than the ACEIs group. Attempts were made to minimise potential confounders' influence by applying balanced inclusion and exclusion criteria (e.g., minimising the duration of RAS inhibitor therapy and excluding patients with ESRD, those consuming corticosteroids or birth control pills, patients with anemia and pregnant patients) when selecting the participants. This allowed better control of remaining confounding factors, such as sex, age, smoking habit, blood pressure, diabetes duration, antidiabetic therapy, and medication adherence. We conducted bivariate analysis to select covariates prior to multivariate analysis. Only covariate with *p* < 0.05 in analysis with dependent variable will be continued to multivariate logistic regression analyses.

Statistical analysis shows that ACEIs or ARBs did not differ in albuminuria cases ((*p* > 0.05); Table 2). Patients in the study location received RAS inhibitors class as antihypertensive agents based on their diagnosis with hypertension instead of albuminuria. This observation is in line with the previous meta-analysis, which stated that ACEIs and ARBs did not significantly differ in their effect to lower urinary protein excretion in hypertensive patients [19]. Even though the two drug classes affect different biochemical pathways, their clinical effects are similar [19]. First-line therapies prescribed for hypertensive patients include thiazide diuretics, dihydropyridine calcium channel blockers, ACEIs, and ARBs. It is recommended to increase the dosage of the first drug or to add a second drug of a different class among the first-line recommendations if optimum blood pressure could not be achieved or maintained. The third drug among the first-line recommendations is titrated two drugs combination could not achieve target blood pressure [20]. If after one month of treatment the optimum blood pressure cannot be attained and maintained, it is recommended to increase the dose of the first drugs or add another drug from other classes in the first-line recommendations. If the combination of two drugs could not achieve target blood pressure, titrate a third drug from first-line options [20]. The target of antihypertensive pharmacological therapy is to reduce blood pressure or maintain blood pressure during the treatment period. Therefore, antihypertensive agents were given based on patients' clinical conditions. A previous study supports the intensive decrease of blood pressure to prevent kidney failure, so the number of antihypertensive agents increases in patients with persistent hypertension [21]. The hypothesis supporting the intensive blood pressure lowering approach to decrease albuminuria has encouraged the medical practitioner to prescribe numerous antihypertensive drugs [21]. Cross-sectional data highlights the prescribing pattern by the medical practitioner in the study location. This results in a wide variety of hypertensive drugs received by the patients but still following the recommendation of the guidelines and national formulary [14, 20].

We observed that patients with uncontrolled diastolic blood pressure more often suffer from albuminuria than patients having controlled diastolic blood pressure (Table 2). This result supports a previous study which showed that diastolic blood pressure above 80 mmHg increases the risk of albuminuria [22]. Other studies demonstrated that targeting low target blood pressure (<120/70 mmHg) reduces proteinuria, even during concurrent glycaemic control strategies, and blood pressure target 140/90 mmHg might be too high for renal protection in diabetic patients [23].

The observation of fewer female patients with albuminuria (Table 2) may imply a higher nitric oxide (NO) levels in female patients, possibly associated with the generally lower oxidative stress levels in the female [25]. High oxidative stress levels in diabetic males were related to diabetic nephropathy progression and marked by reductions in eGFR and increases in ACR.

One of the several significant adverse events related to RAS inhibitor therapy is hyperkalemia [24]. Statistical analysis in the present study showed that ACEI or ARB treatment did not affect hyperkalemia (*p* > 0.05; Table 3). Excretion dysfunction, transcellular shift, increase in potassium intake, and pseudo-hyperkalemia are other factors that might increase serum potassium levels. Hyperkalemia mediated by renal dysfunction may be due to the dysfunction of one or several processes, including the distant nephron flow rate, the intensity of aldosterone secretion, and potassium secretion pathways [25]. Female patients were found to present with hyperkalemia less often than males (*p* < 0.03; Table 3). This result supports past research that females have lower potassium concentration than males [26].

Patients with moderate medication adherence have fewer hyperkalemia cases than patients with high medication adherence, but not significant (*p*=0.136; Table 3). Medication adherence in this study was related to the patient's adherence to both antidiabetic and antihypertensive treatments. Low medication adherence is known to lead to impaired glycaemic and blood pressure control and increases the incidence of adverse events [27, 28]. Patients with uncontrolled systolic blood pressure less often show hyperkalemia than patients with better control (*p*=0.103; Table 3). This result shows that routine use of medication may increase hyperkalemia in T2DM patients on ACEIs or ARBs.

Overall, the results of the present study showed that sex and diastolic blood pressure control affect the number of albuminuria cases, whereas sex, systolic blood pressure control, and medication adherence affect the number of cases of hyperkalemia.

The present study had a cross-sectional design that does not allow far-reaching recommendations about the longitudinal effects of the examined treatments. Future cohort studies will be needed to address these issues. We would suggest a more extensive data collection regarding smoking habits, increasing the number of patients recruited, and recruitment of patients from multiple locations. Nevertheless, the present study provided initial insights into the effects of ACEIs versus. ARBs on albuminuria and hyperkalemia in hypertensive T2DM patients.

## 5. Conclusions

This research demonstrates that treatments with ACEIs and ARBs have similar effects on albuminuria and hyperkalemia, whether confounding variables are corrected for or not.

## Figures and Tables

**Table 1:** Anthropometric, sociodemographic, lifestyle, clinical, and pharmacological therapy characteristics of T2DM patients on ACEIs or ARBs.

Characteristics	ACEIs	ARBs	*p* value
	*N* = 57 (42.5)	*N* = 77 (57.5)	
*Sex*			0.518^a^
Male	30 (52.6)	35 (45.5)	
Female	27 (47.4)	42 (54.5)	
*Age (years)*	58.07 ± 8.09	59.04 ± 8.25	0.499^c^
Categorical			0.835^b^
31–40 years	2 (3.5)	1 (1.3)	
41–50 years	6 (10.5)	8 (10.4)	
51–60 years	29 (50.9)	38 (49.4)	
≥60 years	20 (35.1)	30 (39.0)	
*BMI (kg/m* ^*2*^)	26.85 ± 4.11	27.06 ± 4.46	0.782^c^
Categorical			0.008^b^^*∗*^
Underweight	1 (1.8)	− (0.0)	
Normal	9 (15.8)	19 (24.7)	
Overweight	13 (22.8)	4 (5.2)	
Obesity I	18 (31.6)	38 (49.4)	
Obesity II	16 (28.1)	16 (20.8)	
*Occupation*			1.000^a^
Employed	14 (24.6)	20 (26.0)	
Unemployed	43 (75.4)	57 (74.0)	
*Education*			0.422^b^
Uneducated	—(0.0)	2 (2.6)	
Elementary school graduates	5 (8.8)	5 (6.5)	
Junior high school graduates	10 (17.5)	7 (9.1)	
Senior high school graduates	21 (36.8)	33 (42.9)	
College graduate	21 (36.8)	30 (39.0)	
*Smoking habit*			0.399^b^
Smokers	6 (10.5)	6 (7.8)	
Nonsmokers	48 (84.2)	62 (80.5)	
Ex-smokers	3 (5.3)	9 (11.7)	
*Duration of type 2 diabetes (years)*	10 (1–30)	10 (2–46)	0.554^d^
Categorical			0.296^b^
≤5 years	13 (22.8)	11 (14.3)	
>5 years	44 (77.2)	66 (85.7)	
*Systolic blood pressure (mmHg) (n−102)*	133 (90–193)	137 (97–205)	0.309^d^
Categorical			0.141^a^
≤140 mmHg	29 (67.4)	30 (50.8)	
>140 mmHg	14 (32.6)	29 (49.2)	
*Diastolic blood pressure (mmHg) (n* *=* *102)*	79 (54–105)	79 (45–96)	0.690^d^
Categorical			0.505^a^
≤90 mmHg	35 (81.4)	52 (88.1)	
>90 mmHg	8 (18.6)	7 (11.9)	
*HbA1c (%)*	7.3 (5.5–11.7)	7.5 (5.1–13.3)	0.466
Categorical			0.485^a^
≤7.0%	25 (43.9)	28 (36.4)	
>7.0%	32 (56.1)	49 (63.6)	
*LDL cholesterol (mg/dL)*	115 (58–190)	121 (47–264)	0.366^d^
Categorical			0.926^a^
<100 mg/dL	15 (26.3)	22 (28.6)	
≥100 mg/dL	42 (73.7)	55 (71.4)	
*ACR (mg/g)*	47.6 (3.20–4060.5)	50.80 (3.8–5504.6)	0.345^d^
Categorical			0.970^a^
≤30 mg/g	24 (42.1)	31 (40.3)	
>30 mg/g	33 (57.9)	46 (59.7)	
*eGFR (mL/min/1.73 m* ^*2*^)	71.41 ± 19.93	64.08 ± 20.50	0.040^c^^*∗*^
Categorical			0.051^a^
≥60 mL/min/1.73 m^2^	40 (70.2)	40 (51.9)	
30–59 mL/min/1.73 m^2^	17 (29.8)	37 (48.1)	
*Serum potassium (mmol/L)*	4.9 (3.62–6.50)	4.9 (2.97–9.30)	0.780^d^
Categorical			0.860^a^
<5.5 mmol/L	46 (80.7)	60 (77.9)	
≥5.5 mmol/L	11 (19.3)	17 (22.1)	
*Diabetes therapy*			0.569^b^
Oral antidiabetic agents	22 (38.6)	26 (33.8)	
Insulin	15 (26.3)	17 (22.1)	
Combination therapy	20 (35.1)	34 (44.2)	
*Oral antidiabetic agents (n* *=* *102)*			0.616^b^
Biguanides	17 (29.8)	24 (31.2)	
Sulfonylurea	2 (3.5)	7 (9.1)	
Α-glucosidase inhibitors	0 (0.0)	1 (1.3)	
Combination	23 (40.4)	28 (36.4)	
*Hypertension therapy*			0.110^b^
ACEIs/ARBs monotherapy	12 (21.1)	16 (20.8)	
ACEIs/ARBs + 1 non-RAS inhibitors	30 (52.6)	33 (42.9)	
ACEIs/ARBs + 2 non-RAS inhibitors	8 (14.0)	21 (27.3)	
ACEIs/ARBs + 3 non-RAS inhibitors	4 (7.0)	7 (9.1)	
ACEIs/ARBs + 4 non-RAS inhibitors	3 (5.3)	− (0.0)	
*ACEIs/ARBs usage duration (months)*	6 (2–54)	16 (2–61)	*p* < 0.001^*d∗*^
Categorical			*p* < 0.001^*b∗*^
≤3 months	23 (40.4)	5 (6.5)	
3–6 months	9 (15.8)	9 (11.7)	
>6 months	25 (43.8)	63 (81.8)	

ACEIs, angiotensin converting enzyme inhibitors; ARBs, angiotensin II receptor blockers, ACR, albumin to creatinine ratio; eGFR, estimated glomerular filtration rate (CKD-EPI equation). ^a^Continuity correction; ^b^Pearson chi-square; ^c^independent t-test; ^d^Mann–Whitney test; denominator for each characteristic is number in respective groups; categorical data presented as *n* (%), continuous data presented in mean ± SD or median (min-max); ^*∗*^statistically significant.

**Table 2:** Factors affecting the cases of albuminuria in T2DM patients on ACEIs or ARBs.

	Variable	*p* value	OR	95% CI	
				Upper	Lower
Crude model	RAS inhibitor				
	ACEI	Reference			
	ARB	0.830	1.079	0.538	2.164
Adjusted model 1	RAS inhibitor				
	ACEI	Reference			
	ARB	0.849	1.085	0.466	2.527
	Sex				
	Male	Reference			
	Female	0.069	0.463	0.202	1.063
	Diastolic blood pressure				
	Controlled	Reference			
	Uncontrolled	0.045^*∗*^	4.964	1.033	23.863
Adjusted model 2	Sex				
	Male	Reference			
	Female	0.068	0.461	0.201	1.058
	Diastolic blood pressure				
	Controlled	Reference			
	Uncontrolled	0.046^*∗*^	4.897	1.026	23.366

ACEIs, angiotensin converting enzyme inhibitors; ARBs, angiotensin II receptor blockers. ^*∗*^Statistically significant.

**Table 3:** Factors affecting the cases of hyperkalemia in T2DM patients on ACEIs or ARBs.

	Variable	*p* value	OR	95% CI	
				Upper	Lower
Crude model	RAS inhibitor				
	ACEI	Reference			
	ARB	0.696	1.185	0.506	2.772
Adjusted model 1	RAS inhibitor				
	ACEI	Reference			
	ARB	0.662	1.282	0.420	3.911
	Sex				
	Male	Reference			
	Female	0.076	0.358	0.115	1.115
	Smoking habit				
	Nonsmokers	Reference			
	Ex-smokers	0.116	3.717	0.724	19.087
	Systolic blood pressure				
	Controlled	Reference			
	Uncontrolled	0.122	0.398	0.124	1.281
	Medication adherence				
	High adherence	Reference			
	Moderate adherence	0.104	0.387	0.124	1.214
Adjusted model 2	Sex				
	Male	Reference			
	Female	0.028^*∗*^	0.299	0.102	0.877
	Systolic blood pressure				
	Controlled	Reference			
	Uncontrolled	0.103	0.401	0.134	1.202
	Medication adherence				
	High adherence	Reference			
	Moderate adherence	0.136	0.443	0.152	1.291

RAS, renin-angiotensin-aldosterone system; ACEIs, angiotensin converting enzyme inhibitors; ARBs, angiotensin II receptor blockers. ^*∗*^Statistically significant.

## Data Availability

The data used in the study are available upon request to the corresponding author.
